# Dynamics of oropharyngeal structures during respiration with the volume-altered tongue base of minipigs

**DOI:** 10.1590/1678-7765-2025-0722

**Published:** 2026-06-12

**Authors:** Nino Geradze, Doris Haydee Rosero Salazar, Phuc Hoang Nguyen, Zi-Jun Liu

**Affiliations:** 1 University of Washington Department of Orthodontics Seattle USA University of Washington, Department of Orthodontics, Seattle, USA; 2 Universidad Icesi Department of Pharmaceutical Sciences Faculty of Engineering, Design, and Applied Sciences Cali Colombia Universidad Icesi, Department of Pharmaceutical Sciences, Faculty of Engineering, Design, and Applied Sciences, Cali, Colombia

**Keywords:** Oropharynx, Tongue Base, Videofluoroscopy, Respiration, Minipig

## Abstract

**Objective:**

This study aimed to analyze the functional adaptations of tongue base volumetric enlargement and reduction on the kinematics of oropharyngeal structures during respiration in a minipig model.

**Methodology:**

Six same-sex sibling pairs of 8-to-9-month-old Yucatan minipigs were studied. Of each pair, one was diet-induced obese with a BMI>50 (obesity-associated volume enlargement) while the other was normal-weight and underwent partial ablation of the tongue base volume (volume-reduction). Real-time X-ray video fluoroscopic images (30 frames/s) were recorded under sedation, at baseline (before surgery) and 5 weeks postoperatively. Selected landmarks of the soft palate, epiglottis, tongue base, and pharyngeal wall were digitized frame by frame for 25–30 respiratory cycles. Directional movements and distance changes of these oropharyngeal structures were analyzed within the defined coordinate system using video-analysis software. Correlations between areas of airway spaces during respiration and biometric measurements of the dissected tongue at week 5 were included.

**Results:**

During respiratory phases, movement distances of the soft palate and the pharyngeal wall were significantly larger in the volume-reduced group than in the volume-enlarged group at baseline (p<0.05). These moving distances were also larger compared to those of the volume-enlarged group at both time points. Similarly, distance changes during inspiration between structures (soft palate-pharyngeal wall, soft palate-tongue base, and epiglottis-pharyngeal wall) were significantly larger at week 5 than at baseline (p<0.05) in both groups. Positive correlations during respiratory phases were detected between tongue volume and velopharyngeal width in the volume-reduced group (r=0.86, p<0.05). Negative correlations occurred between retromolar space area and tongue thickness in the volume-enlarged group (r=−0.9, p<0.05).

**Conclusions:**

These results suggest that oropharyngeal spatial dynamics are enhanced in the tongue base volume-reduced minipigs, with altered moving patterns during respiration. In contrast, smaller distance changes observed over time in the volume-enlarged group suggest continuous airway narrowing and potential restriction of airway dynamics.

## Graphical abstract

**Figure:**
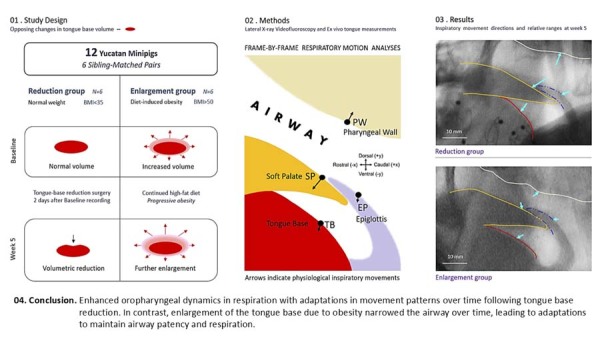

## Introduction

The oropharynx is a dynamic passage where highly synchronized movements of the tongue base, soft palate, epiglottis, and pharyngeal wall maintain airway patency during respiration.^1^ However, volumetric changes of these structures may alter respiratory airflow and, consequently, affect peripheral tissue perfusion. This is particularly evident in clinical conditions such as obstructive sleep apnea (OSA), where obesity-related enlargement of soft tissues increases the risk of airway collapse and disrupts normal respiratory dynamics.^2^ Diminished respiratory movements observed clinically and in several imaging and functional studies suggest additional tissue and neural control impairments that compromise airway stability.^3,4^

Our previous studies in the normal minipig model (non-obese, non-surgical control group) showed specific functional spatial configurations of oropharyngeal structures during respiration.^5,6^ Overall, the normal tongue base in this model showed increased length (dorsally and ventrally), thickness (anteroposterior and bilateral), and width (anterior region, dorsally and ventrally) during the inspiratory phase of respiration. Similarly, our recent publication showed distinctive opposite dimensional deformations of the intact and volumetrically altered tongue base when tongue muscles and the hypoglossal nerve were electrically stimulated.^7^ For instance, controls exhibited thinning, whereas the experimental groups exhibited widening and thickening when the nerve and the genioglossus muscle were stimulated, respectively. Respiratory rates also improved in the volumetrically enlarged tongue base while tidal volume increased in the volume-reduced minipig models.

Surgical volumetric reduction of the tongue base, or partial glossectomy, has been clinically performed to treat tongue cancers, macroglossia, and obstructive sleep apnea (OSA), among other conditions.^8^ However, the mechanisms by which this procedure affects respiratory physiology and oropharyngeal dynamics remain unclear. In the present study, we examined how experimentally induced volume alterations of the tongue base, either enlargement via obesity or reduction via surgical controlled ablation, affected oropharyngeal dynamics and respiratory airflow profiles in the Yucatan minipig model. The model was chosen because 1) the minipig’s tongue resembles that of humans in both shape and size, and the tongue base strongly influences oropharyngeal airway dynamics through its contact with the soft palate in both species; 2) the minipig’s oropharyngeal airway is comparable to that of humans, with a similar microstructural architecture composed of the same tissue types;^9^ 3) the size and shape of the hyoid bone of the minipig conform generally with that of adult humans;^10^ and 4) the minipig provides adequate size for instrumentation and therapeutic intervention.^11,12^ Therefore, this model closely mirrors human oropharyngeal anatomy in tongue base–pharyngeal wall proportions, soft palate mechanics, and airway curvature,^13,14^ making it well suited to address the aim of the present study. Here, we examined how experimentally induced volume alterations of the tongue base—either enlargement via obesity or reduction via surgical controlled ablation—functionally affect oropharyngeal respiratory dynamics and airflow profile in the Yucatan minipig model. These two experimental pathological models are highly clinically relevant, and research on muscular/lingual damage, remodeling, and regeneration are specific aims of the main design, the results of which will be published elsewhere. In the present study, we hypothesized that tongue base enlargement may restrict the respiratory movements of oropharyngeal structures, whereas tongue base reduction may increase their dynamic movements, thereby contributing to improved airway patency. Understanding these changes in oropharyngeal structures may help clinicians better evaluate outcomes of volumetric alterations of the tongue base. This will contribute to improving current treatments for various clinical conditions, including macroglossia and sleep breathing disorders such as OSA.

Based on these considerations, the question addressed in this study is: how do two distinct conditions of tongue-base volume—obesity-associated volume enlargement and surgical reduction—influence respiratory kinematics and inter-structural spatial relationships between oropharyngeal structures? By addressing this question, we aimed to clarify how volumetric alterations of the tongue base impact the temporal and spatial airway dynamics.

## Methodology

### Animals

A total of six same-sex sibling pairs (8–9-month-old, three of each sex, n = 12, Premier BioSource, Ramona, CA) were studied. Each pair was randomly assigned to an experimental group. The reduction group consisted of minipigs with normal body weight (BMI < 35) that underwent partial surgical volumetric reduction of the tongue base. The enlargement group consisted of obese minipigs (BMI > 50) with a volume-enlarged tongue base, achieved by intentional overfeeding with a high-fat diet for 10+ weeks before delivery, as obesity is a recognized contributing factor to acquired *macroglossia.*^2^ These sample sizes in each group were justified by a > 80% power estimate with a conservative 1.4-to-1.6-fold ratio of means for detecting a difference between these benchmark measures at a 5% significance level based on a two-tailed Mann–Whitney test.^15^ Upon arrival, the minipigs were acclimated to the new environment for 5–7 days. The enlargement group was continuously fed a high-fat diet *ad libitum* provided by the vendor to maintain the obese condition, while the reduction group was fed regular chow pellets twice daily. Surgical implantation of ultrasound crystals in the tongue base and multiple metal markers in the upper and lower alveolar bones was performed 7–10 days after arrival, as described previously.^5,6^

All procedures were approved by the Institutional Animal Care and Use Committee (IACUC, Protocol# 3393-05) of the University of Washington and were conducted in accordance with the National Institutes of Health (NIH) guidelines for the care and use of laboratory animals.

### Baseline X-ray Videofluoroscopy

One week after ultrasonic crystal implantation surgery, as described elsewhere,^6^ baseline X-ray videofluoroscopy was performed (OEC 9900 Elite X-ray system, General Electric Co., Evendale, OH). Imaging procedures are also previously described in detail elsewhere.^5^ In brief, the sedated minipig was positioned prone for lateral X-ray videofluoroscopy with a field of view (FOV) on the oropharyngeal region during spontaneous respiration for 2 minutes at 30 frames per second. All X-ray videos were recorded simultaneously with respiratory airflow through a mouth mask connected to a sensor (TSD160A-TSD237F) and interfaced with the MP150 system (Biopac Co, Goleta, CA) to characterize respiratory phases and airflow parameters.

### Volumetric Reduction Surgery and Terminal Imaging

Two days after the baseline recording, minipigs in the reduction group underwent partial surgical tongue base ablation using controlled radiofrequency, or coblation, as described in clinical settings.^16^ The coblation technique was used to remove 1.20 ± 0.48cm^3^ (4–8%) of the original tongue base volume, with inter-animal variations.^7,17^ This controlled ablation surgery was performed under direct visualization with the mouth wide open and the tongue body pulled down and forward to expose the two circumvallate papillae as the anterior border of the tongue base. The coblation site was the center of the tongue base, 10mm posterior to the papillae, with an extent of 15–20mm and a depth of 10–15mm, and the ablated tissue was preserved in 0.9% saline. No intervention was carried out in the enlargement group, as obesity has been demonstrated to be a major cause of acquired macroglossia.^2^ Five weeks following the reduction surgery, both experimental groups received terminal X-ray videofluoroscopic imaging using the same procedures as at baseline.

### Imaging Digitization and Analysis

The procedure for image digitization followed previously established methods.^5^ In brief, the traced anatomical structures included: 1) posterior 1/3 of the ventral surface of the soft palate; 2) anterior 1/3 of the epiglottis; 3) midpoint of the dorsal surface of the tongue base; and 4) posterior 1/3 of the dorsal pharyngeal wall.

**Figure 1:**
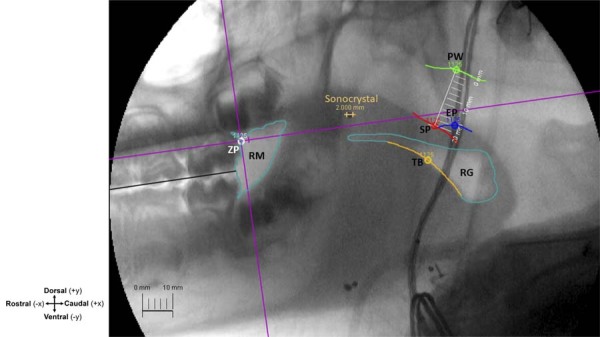
Preset coordinate system and tracing points on each oropharyngeal structure. The X-axis is defined as a line extending across the alveolar ridge of the upper molars, parallel to the occlusal plane (arrow), and the Y-axis is perpendicular to the X-axis, passing through the distal surface of the last upper molar. The intersection between the X- and Y-axes is used as the zero point (ZP). Digitized markers (small circles) for the soft palate (SP), tongue base (TB), epiglottis (EP), and pharyngeal wall (PW) were traced during inspiration and expiration in the coordinate system. The system was calibrated using the standard 2-mm size of the ultrasonic crystals implanted before baseline recordings. Blue lines indicate areas of the retromolar (RM) and retroglossal (RG) regions. The velopharyngeal width was measured as the linear distance between the points of PW and SP (ruler). The double-arrowed cross (lower left) indicates anatomical orientations for all structural tracing. Scale: 10mm.

After defining a preset X-Y coordinate and establishing a zero point (reference point), the four oropharyngeal structures were digitized frame by frame over 25–30 respiratory cycles using Tracker 6.2.0 video-analysis tool (**Figure 1**). The previously implanted 2-mm round ultrasonic crystals served as calibration markers. Each digitized point was expressed in X and Y values relative to the zero point in the coordinate.

Respiratory phases were identified from synchronized respiration recordings and defined as inspiration, expiration, and post-expiratory pause. Minipigs are predominantly nasal breathers, and the downward movement of the soft palate at the start of respiration served as an indicator for tracing structural changes during inspiration. Conversely, the opposite movement served as the sign for expiratory phases. Respiratory airflow recordings by the Biopac system confirmed increased airflow velocity and decreased airflow pressure during inspiration.

Once all tracings were completed, the Euclidean distance formula ( $d=\sqrt{}((x2-x1{)}^{2}+(y2-y1{)}^{2}$ )^18^ was used to calculate the actual moving distance of each digitized point in relation to the X-Y coordinate. The orientations of these movements were further analyzed and defined as rostral (anterior), caudal (posterior), dorsal (upward), and ventral (downward) directions. Analyses were performed for both the moving distance of individual structures (structural) and the moving distance changes between two given structures (inter-structural).

In addition, by detecting maximum inspiration and maximum expiration via the downward (inspiration) and upward (expiration) movements of the soft palate, velopharyngeal width and the areas of the retroglossal and retromolar spaces during inspiration and expiration were separately measured in X-ray frames using ImageJ (Bethesda, MD). The velopharyngeal width was defined as the width of the space dorsal to the midpoint of the soft palate (velopharynx), and the retroglossal and retromolar space areas are marked in Fig. 1 (RM and RG). These measurements at week 5 were further correlated with biometric measurements (volume, length, width, thickness, and weight) of the whole tongues and tongue bases dissected after the terminal imaging.

The tongue base was defined as the region between the two circumvallate papillae anteriorly and the posterior border of the tongue. Length was measured from the posterior border to the tip of the tongue. Thickness was measured dorso-ventrally at the thickest point of the tongue. Width was measured from one lateral edge to the other over the circumvallate papilla on the dorsal surface of the tongue. For volume, either the entire tongue or the tongue base was submerged in 0.9% saline solution, and the increase in solution volume in mL was recorded as the measurement. For weight, each structure was placed on a tray on a scale and measured in grams.

### Reliability Tests and Statistics

The inter- and intra-examiner reliabilities of the measurement methods were examined. First, 10–15 seconds of data were randomly selected and digitized by one investigator (NG), then independently measured by a second investigator (PHN). A paired t-test revealed no significant differences between digitized measurements by the two investigators. The intraclass correlation coefficient was 0.970, p=0.000. Second, randomly selected frames were digitized and measured at a 10-day interval by the first investigator (NG). The error calculated using Dahlberg’s formula was 0.02mm (below 0.5mm).^19^

All raw values of movement distances were normalized to neck circumference, measured over the thyroid cartilage, to account for size differences among animals. Neck circumference is a representative measure of growth over time in the oropharyngeal region. Data normalization was used to standardize size differences between normal and obese minipigs at baseline and growth over the experimental period.

The Kolmogorov–Smirnov and Shapiro–Wilk tests of normality of the respiration data showed p < 0.05. Therefore, the non-parametric Kruskal–Wallis test followed by pairwise multiple comparisons (Mann–Whitney U-test) was applied to assess significant changes in the movements of all structures between the two groups at each time point, as well as between time points (baseline and week 5) within each group.

Pearson’s r correlations were determined for the last time point between X-ray measurements during inspiration and expiration and biometric measurements of weights, volumes, and dimensions of the whole tongue and the tongue base. These data showed normal distribution, and one-way ANOVA followed by Tukey post-hoc was used for comparisons between groups and time points. A significance level of p < 0.05 was set for all comparisons. All statistical analyses were performed using SPSS version 19.0 (IBM).

## Results

### Body sizes and growth

**Table 1** summarizes the measurements of body mass index (BMI), body length, and neck circumference for both groups across the two time points when the X-ray videofluoroscopy recordings took place. In the volume-reduced group, these three parameters were significantly different between the two time points (p < 0.001). In the volume-enlarged group, only body length was significantly different between baseline and week 5 (p < 0.01). All parameters were significantly different between groups at both time points (p < 0.001).

**Table 1:** Average ± SD of body sizes for each experimental group and timepoint.

Group	Body Mass Index		Body Length (cm)		Neck Circumference (cm)	
	Baseline	Week 5	Baseline	Week 5	Baseline	Week 5
Reduction	33 ± 3.1ª*	38 ± 3.1ª*	104 ± 4.5ᵇ*	111 ± 3.1ᵇ*	58 ± 3.0ᶜ*	63 ± 3.0ᶜ*
Enlargement	56 ± 6.6*	60 ± 3.7*	116 ± 6.7ᵇ*	124 ± 5.5ᵇ*	75 ± 3.4*	75 ± 2.3 *

a : significant differences between timepoints in body mass index.

b : significant differences between timepoints in length.

c : significant differences between timepoints in neck circumference.

*: significant differences between groups.

Body length measured from the snout to the base of the tail.

### Respiratory dynamics

The respiratory rates per minute were 27.7 ± 9.5 at baseline and 32.5 ± 2.1 at week 5 in the volume-reduced group, and 25.5 ± 6.5 at baseline and 18.1 ± 2.8 at week 5 in the volume-enlarged minipigs. No significant differences were detected between the two groups or between the two time points. These respiratory rates exhibited opposite trends over time, increasing in the volume-reduced group and decreasing in the volume-enlarged group.

**Figure 2:**
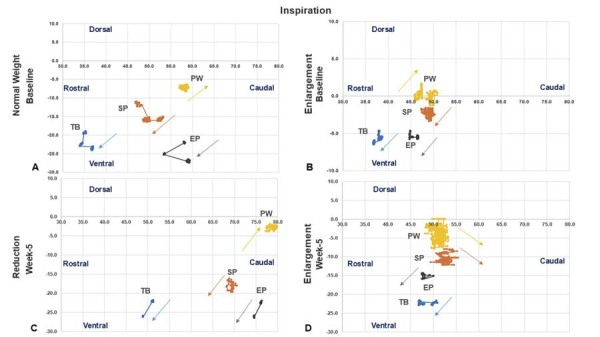
Respiratory movements in the coordinate system during inspiration at baseline and week 5 in both experimental groups. TB: tongue base; SP: soft palate; EP: epiglottis; PW: pharyngeal wall. Averages of 25–30 respiratory cycles per minipig were included in the construction of the superposed digitized points. The arrows indicate the direction of movement for each structure.

**Figure 3:**
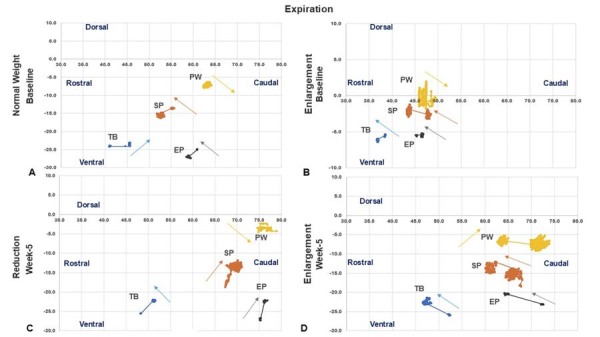
Respiratory movements in the coordinate system during expiration at baseline and week 5 in both experimental groups. TB: tongue base; SP: soft palate; EP: epiglottis; PW: pharyngeal wall. Averages of 25–30 respiratory cycles per minipig were included in the construction of these graphs. The arrows indicate the direction of movement for each structure.

In the volume-reduced group, the movement direction of the soft palate on the X-Y coordinate was rostral-ventral during inspiration at both time points, and rostral-dorsal and caudal-dorsal during expiration at baseline and week 5, respectively (**Figures 2 and 3**). These directions in the volume-enlarged group were rostral-ventral at baseline during both respiratory phases and changed to caudal-ventral and rostral-dorsal in inspiration and expiration, respectively, at week 5. Overall, differences were not significant between groups.

The linear distance change of the soft palate during inspiration and expiration in both groups was significantly smaller at baseline compared with week 5 (p < 0.05). The soft palate in the volume-enlarged group showed a shorter distance change during inspiration at week 5 compared to that of the reduction group (**Table 2**).

**Table 2:** Linear distance changes in respiratory phases for each experimental group and timepoint.

			Baseline		Week 5	
	Structure	Group	Inspiration	Expiration	Inspiration	Expiration
**Linear structural distance change**	**SP**	**Reduction**	0.19	0.10	0.54	0.18
			(0.03-0.30) **ª**	(0.03-0.19) **ᵇ**	(0.21-0.66)**ª**	(0.14-0.31) **ᵇ**
		**Enlargement**	0.15	0.11	0.31	0.23
			(0.12-0.18) **ª**	(0.06-0.15) **ᵇ**	(0.09-0.42) **ª**	(0.09-0.24) **ᵇ**
	**EP**	**Reduction**	0.06	0.03	0.07	0.04
			(0.05-0.12)	(0.02-0.08)	(0.03-0.16)	(0.02-0.07)
		**Enlargement**	0.08	0.07	0.02	0.02
			(0.03-0.12)	(0.05-0.08)	(0.01-0.04)	(0.01-0.06)
	**TB**	**Reduction**	0.05	0.04	0.05	0.03
			(0.04-0.16)	(0.02-0.16)	(0.04-0.12)	(0.02-0.05)
		**Enlargement**	0.05	0.06	0.04	0.02
			(0.04-0.05)	(0.03-0.09)	(0.02-0.13)	(0.01-0.45)
	**PW**	**Reduction**	0.15	0.10	0.55	0.21
			(0.03-0.36) **ª**	(0.02-0.27) **ᵇ**	(0.31-0.59) **ª**	(0.16-0.25) **ᵇ**
		**Enlargement**	0.07	0.08	0.21	0.21
			(0.03-0.12) **ª**	(0.03-0.14) **ᵇ**	(0.15-0.46) **ª**	(0.12-0.38) **ᵇ**
**Linear inter-structural distance change**	**SP-TB**	**Reduction**	0.20	0.12	0.51	0.18
			(0.03-0.30) **ª**	(0.03-0.19) **ᵇ**	(0.21-0.64) **ª**	(0.13-0.30) **ᵇ**
		**Enlargement**	0.13	0.07	0.31	0.02
			(0.12-0.15) **ª**	(0.05-0.08) **ᵇ**	(0.19-0.39) **ª**	(0.01-0.45) **ᵇ**
	**EP-TB**	**Reduction**	0.05	0.03	0.08	0.03
			(0.04-0.14)	(0.02-0.12)	(0.01-0.15)	(0.01-0.07)
		**Enlargement**	0.06	0.05	0.02	0.02
			(0.04-0.09)	(0.02-0.06)	(0.01-0.13)	(0.01-0.48)
	**SP-PW**	**Reduction**	0.26	0.14	0.99	0.36
			(0.04-0.45) **ª**	(0.04-0.33) **ᵇ**	(0.48-1.17) **ª**	(0.26-0.51) **ᵇ**
		**Enlargement**	0.16	0.09	0.43	0.36
			(0.15-0.17) **ª**	(0.05-0.12) **ᵇ**	(0.21-0.76) **ª**	(0.18-0.53) **ᵇ**
	**EP-PW**	**Reduction**	0.17	0.09	0.61	0.22
			(0.03-0.36) **ª**	(0.03-0.27) **ᵇ**	(0.31-0.66) **ª**	(0.15-0.29) **ᵇ**
		**Enlargement**	0.11	0.06	0.21	0.20
			(0.10-0.11) **ª**	(0.04-0.07) **ᵇ**	(0.15-0.44) **ª**	(0.11-0.41) **ᵇ**

Notes: Summary of structural changes in Median (Range) of the soft palate (SP), epiglottis (EP), tongue base (TB), pharyngeal wall (PW), including changes between structures or inter-structural. ª Indicates significant differences in inspiration between baseline and week 5. ᵇ Indicates significant differences in expiration between baseline and week 5 by Kruskal-Wallis and U-test. P value < 0.05. Units in millimeters.

During inspiration, the epiglottis in the X-Y coordinate moved rostral-ventral in both groups at both time points. During expiration, the reduction group showed rostral-dorsal movements of the epiglottis at baseline, shifting to caudal-dorsal at week 5. The enlargement group showed rostral-dorsal directions at both time points (**Figures 2 and 3**).

Minor linear distance changes of the epiglottis in both groups were observed at both time points, with no significant differences between the two groups or time points.

The tongue base at baseline and week 5 showed rostral-ventral movements during inspiration in both experimental groups. During expiration, the reduction group showed caudal-dorsal movements at baseline and rostral-dorsal at week 5. The volume-enlarged group exhibited rostral-dorsal directions at both time points (**Figures 2 and 3**).

The linear distance change of the tongue base in the volume-enlarged group was greater during expiration at week 5 than at baseline and those of the volume-reduced group, although no significant differences were detected (**Table 2**).

The movements of the pharyngeal wall in the reduction group were caudal-dorsal during inspiration and caudal-ventral during expiration at both time points. In the volume-enlarged group, these movements were caudal-dorsal and caudal-ventral during inspiration at baseline and week 5, respectively. During expiration, these movements were caudal-ventral and caudal-dorsal at baseline and week 5, respectively (**Figures 2 and 3**).

The linear distance change of the pharyngeal wall was significantly shorter at baseline in both groups and both respiratory phases when compared with week 5 (p < 0.05). These linear changes during inspiration were also larger in the reduction group at week 5 than those of the enlargement group (p < 0.05).

As shown in **Table 2**, inter-structural distance changes between soft palate-tongue base, soft palate-pharyngeal wall, and epiglottis-pharyngeal wall were larger at week 5 than at baseline in both experimental groups and respiratory phases (p < 0.05). All inter-structural changes were larger in the reduction group at baseline in both respiratory phases, and at week 5 during inspiration, compared to those of the enlargement group.

### Correlations between airway spaces and tongue biometrics

As shown in **Table 3**, the volumes of the whole tongue and tongue base at the terminal time points were significantly higher in the volume-enlarged group than in the volume-reduced group (whole tongue p < 0.03; tongue base p < 0.001). Thicknesses of the tongue base were also significantly different between groups (p < 0.02).

**Table 3:** Biometric measurements of the tongue and tongue base for each experimental group. Average ± SD.

Measurement	Group	Whole Tongue	Tongue Base
**Volume (ml)**	**Reduction**	111.7 ± 12.9*	34.8 ± 5.0*
	**Enlargement**	137.0 ± 5.7*	45.0 ± 3.5*
**Length (cm)**	**Reduction**	15.4 ± 1.1	4.1 ± 0.4
	**Enlargement**	16.2 ± 1.3	4.3 ± 0.1
**Width (cm)**	**Reduction**	4.7 ± 0.4	3.8 ± 0.2
	**Enlargement**	4.2 ± 0.5	3.7 ± 0.9
**Thickness (cm)**	**Reduction**	3.1 ± 0.2	3.1 ± 0.3*
	**Enlargement**	3.3 ± 0.3	3.5 ± 0.4*
**Weight (gr)**	**Reduction**	107.7 ± 10.4	35.5 ± 4.8
	**Enlargement**	125.5 ± 17.2	40.6 ± 11.0

*: significant differences between groups

The velopharyngeal width was larger in the volume-enlarged group at baseline than at week 5 and larger than that of the volume-reduced group. This parameter was greatest in the volume-reduced group at week 5, though no significant differences were found (**Table 4**).

**Table 4:** X-ray measurements in respiratory phases for each experimental group and timepoint. Average ± SD.

		Baseline		Week 5	
Measurement	Group	Inspiration	Expiration	Inspiration	Expiration
**Velopharyngeal width (mm)**	**Reduction**	13.9 ± 5.6	10.7 ± 5.6	14.3± 5.9	10.3 ± 5.8
	**Enlargement**	16.6 ± 7.7	11.1 ± 8.6	13.2 ± 1.5	5.9 ± 2.9
**Retroglossal space area (cm**^**2**^**)**	**Reduction**	18.5 ± 9.8	18.3 ± 12.2	15.7 ± 10.0	17.3 ± 10.8
	**Enlargement**	17.3 ± 13.2	14.4 ± 9.6	30.5 ± 24.2	28.7 ± 26.5
**Retromolar space area (cm**^**2**^**)**	**Reduction**	5.9 ± 3.8	5.7 ± 3.7	5.6 ± 2.9	5.4 ± 2.8
	**Enlargement**	8.0 ± 1.0	7.2 ± 0.9	9.9 ± 3.0	9.4 ± 2.9

The areas of the retroglossal and retromolar spaces at baseline were larger in the reduction minipigs than in the enlargement group. In contrast, the areas in the enlargement group were larger than those in the reduction group at week 5. No significant differences were found between the two groups or time points.

**Figure 4:**
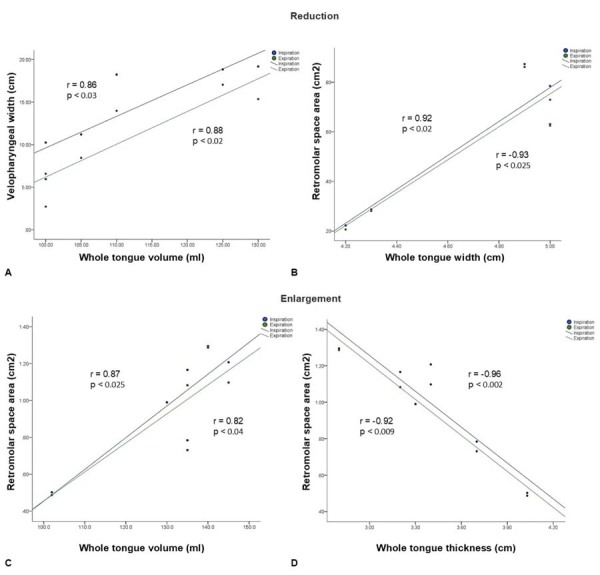
Significant correlations between X-ray measurements and tongue biometrics at week 5 during respiratory phases for both experimental groups.

At week 5, significant positive correlations were detected between velopharyngeal airway width and whole tongue volume in the volume-reduced group in both respiratory phases (inspiration r = 0.86, p < 0.03; expiration r = 0.88, p < 0.02). Additionally, the velopharyngeal width and retromolar space area showed a significant positive correlation with whole tongue width (inspiration r = 0.92, p < 0.02; expiration r = 0.93, p < 0.025, **Figure 4**).

In the volume-enlarged group, significant positive correlations were detected between the retromolar space area and whole tongue volume during respiration (inspiration r = 0.87, p < 0.025; expiration r = 0.82, p < 0.04). In contrast, highly significant negative correlations were seen between the retromolar space area and whole tongue thickness during both respiratory phases (inspiration r = -0.92, p < 0.009; expiration r = −0.96, p < 0.002, **Figure 4**).

## Discussion

The present study analyzed directional movements during respiration in the four pharyngeal structures of normal-weight minipigs before and after induced tongue base volume reduction, and of obese minipigs with enlarged oropharyngeal structures. Functional differences were analyzed between baseline normal and obese conditions and changes over 5 weeks after volumetric reduction of the tongue base and obesity-related oropharyngeal volumetric enlargements. To the best of our knowledge, this is the first longitudinal report on the respiratory dynamics of oropharyngeal structures over time in the context of obesity and volumetric alterations of the tongue base. The outcomes of the present study shed light on the potential mechanism of breathing disorders related to obesity and changes in tongue base volume.

It is worth noting that normal conditions in the present study only occurred at baseline in the reduction group, and results from normal minipigs and functional dynamics in the intact tongue base can be found in our published study.^5^ At baseline, this group was of the same breed but younger compared to those of the present study at the terminal time point. The main limitations of the present study are the lack of a normal control group of the same age at week 5 and the lack of information on injury repair and remodeling after surgical reduction, as well as on obesity development in the tongue base beyond 5 weeks. These drawbacks may restrict the mechanism discussion behind the observed changes in respiratory dynamics of oropharyngeal structures. Nevertheless, histological and immunofluorescence studies of injury repair/myoregeneration and adipose tissue infiltration for these tissue samples are ongoing, which will lead to better exploration of the potential mechanisms and functional adaptations observed in the present study. A complete analysis of tissue remodeling and skeletal muscle regeneration in the injured and adipose-infiltrated tongue bases will be published in an upcoming manuscript.

### Respiratory dynamics of oropharyngeal structures related to normal and obese conditions

Although movements of all structures showed the same directions in both groups at baseline during inspiration, the changes in structural and inter-structural distances were mostly smaller in animals with obesity. This may be attributed to the narrow airway caused by enlargement of the tongue base, soft palate, and pharyngeal wall due to extensive adipose accumulation. This fat tissue infiltration might alter tissue properties, thereby inducing further mechanical consequences that affect respiratory dynamics. A previous study using magnetic resonance elastography reported lower stiffness of oropharyngeal structures in patients with obstructive sleep apnea (OSA) compared to healthy individuals.^20^ Additionally, measurements of neck circumference in humans showed that both young and adult OSA patients had higher values.^21^ This indicates the reliable predictive value of neck circumference in early detection of sleep apnea at various ages.

Multiple factors, including the area of skeletal muscle versus areas of adipose and connective tissues, might result in less efficient directional movements during inspiration and expiration in enlarged oropharyngeal structures. In OSA patients, displacements of the soft palate during quiet breathing under sedation were in the range of 0.27mm–0.61mm throughout respiratory cycles, showing inspiratory narrowing of the velopharyngeal airway.^22^ Presurgical evaluations of OSA patients confirmed increased airflow resistance mainly at the nasopharyngeal airway, which may cause other health issues due to altered tissue perfusion.^23^ The present study confirmed altered dynamics and different directional movement adaptations in the obese model compared to the normal-weight model of the same age.

Further differences between normal and obese animals are observed in the initial positions of the four oropharyngeal structures in both respiratory phases. These four structures spread more widely rostrally and caudally in normal-weight animals than in those with obesity, and this pattern persisted over time. This specific feature in obese minipigs may imply that the physical load of adipose tissue in oropharyngeal structures could lead to a certain degree of airway restriction or respiratory failure.^24^

### Respiratory dynamics of oropharyngeal structures related to volume-reduced and volume-enlarged tongue base

The transoral surgical partial ablation of the tongue base creates defects in areas close to the vallecula and involves muscle resection with some impairment of muscle attachments.^25^ After reduction surgery, inflammation and wound healing may result in scar tissue formation and functional impairment.^26,27^ Scar tissue formation in the tongue base may induce changes in directional movements of the soft palate and epiglottis in the reduced group during expiration, as evidenced by directional shifts from rostral at baseline to caudal at week 5, whereas the tongue base moved from caudal to rostral.

The epiglottis and tongue base are closely related through the glossoepiglottic folds and vallecula, and this region includes attachments of extrinsic tongue muscles surrounded by pharyngeal muscles.^28^ This anatomical relationship may explain the altered directional movements of the epiglottis opposing those of the tongue base during respiration. Such coordination likely induces a more open airway given the relation of both structures with the soft palate and pharyngeal wall, most of which were wider at week 5 post-surgery. Interestingly, all four oropharyngeal structures shifted caudally 5 weeks after surgery (compare Figs. 3A and 3C). This phenomenon suggests that the reduced volume of the tongue base may modify the initial positions of the oropharyngeal structures during respiration, which could be beneficial for airway patency.

In the enlargement group, atypical directional movements of the pharyngeal wall were observed at week 5, opposing those observed at baseline during both respiratory phases. During inspiration, the pharyngeal wall moved ventrally rather than dorsally, following the movement of the soft palate. This may narrow the airway and impair inspiratory efforts with a prolonged respiratory cycle. In patients with higher BMI, narrowing of the pharyngeal passage due to glossal fat accumulation may induce changes in airflow dynamics that could negatively impact peripheral perfusion.^29^

Additionally, the enlargement group showed consistent directional movements of the tongue base and epiglottis over time through both respiratory phases. These movements of the tongue base, epiglottis-tongue base, and soft palate-tongue base were also greater during expiration at the terminal time point. These findings may reflect compensatory mechanisms to improve airflow dynamics, airway patency, and tissue perfusion. However, functional impairment and increased airflow resistance due to a narrower airway passage may further deteriorate oropharyngeal muscle activities, leading to less efficient compensatory mechanisms over time.

### Association of the sizes of the tongue and oropharyngeal airway spaces

In this study, the velopharyngeal width and retromolar space appeared to undergo substantial changes in relation to the altered biometrics of the whole tongue, such as volumes, widths, and thickness, over time. The decrease in velopharyngeal width in the enlargement group over time might be influenced not only by the volume-enlarged tongue base but by overall obesity. Conversely, the increase in this parameter in the volume-reduced group after surgery strongly supports that the configuration of the soft palate is likely modulated by the size of the tongue.

Positive correlations may indicate adaptations that improve airway patency or maintain airflow demands to compensate for high airflow resistance in enlarged oropharyngeal structures. Conversely, negative correlations may indicate a potential risk of poor adaptation due to smaller airway spaces, particularly as tongue thickness increases. The obesity condition in the present study ranged from moderate to severe, without signs of morbidity or functional limitations of the minipigs in daily activities. Yet they showed airway alterations associated with oropharyngeal structure enlargements that might worsen their respiratory function due to continued fat accumulation narrowing airway spaces. However, despite the significant associations between sizes of the tongue and oropharyngeal airway spaces, these reveal certain exploratory, rather than causal, relationships. We expect the results from our ongoing histological and immunofluorescence study to elucidate causal relationships between these observed changes in respiratory dynamics of oropharyngeal structures in relation to the volumetrically reduced and enlarged tongue bases.

## Conclusion

Volumetric alterations of the tongue base significantly influence the respiratory dynamics of oropharyngeal structures during both inspiration and expiration. Reduction of the tongue base volume alters directional movements of the tongue base, soft palate, and epiglottis during expiration. It also increases the movement distances primarily of the soft palate and pharyngeal wall over time following reduction surgery. In contrast, enlargement of the tongue base alters dynamics mostly during inspiration and shows smaller intra- and inter-structural distance changes in comparison to those of the reduction group. Overall, while the reduction group showed more active respiratory dynamics, enlargement of the tongue base and other oropharyngeal structures could have a negative impact on airway patency during respiration.
